# Biosynthesis of organic photosensitizer Zn-porphyrin by diphtheria toxin repressor (DtxR)-mediated global upregulation of engineered heme biosynthesis pathway in *Corynebacterium glutamicum*

**DOI:** 10.1038/s41598-018-32854-9

**Published:** 2018-09-27

**Authors:** Young Jin Ko, Young-Chul Joo, Jeong Eun Hyeon, Eunhye Lee, Myeong-Eun Lee, Jiho Seok, Seung Wook Kim, Chulhwan Park, Sung Ok Han

**Affiliations:** 1 Department of Biotechnology, Korea University, Seoul, 02841 Republic of Korea; 2 Department of Chemical and Biological Engineering, Korea University, Seoul, 02841 Republic of Korea; 3 Department of Chemical Engineering, Kwangwoon University, Seoul, 01897 Republic of Korea

## Abstract

Zn-porphyrin is a promising organic photosensitizer in various fields including solar cells, interface and biomedical research, but the biosynthesis study has been limited, probably due to the difficulty of understanding complex biosynthesis pathways. In this study, we developed a *Corynebacterium glutamicum* platform strain for the biosynthesis of Zn-coproporphyrin III (Zn-CP III), in which the heme biosynthesis pathway was efficiently upregulated. The pathway was activated and reinforced by strong promoter-induced expression of *hemA*^*M*^ (encoding mutated glutamyl-tRNA reductase) and *hemL* (encoding glutamate-1-semialdehyde aminotransferase) genes. This engineered strain produced 33.54 ± 3.44 mg/l of Zn-CP III, while the control strain produced none. For efficient global regulation of the complex pathway, the *dtxR* gene encoding the transcriptional regulator diphtheria toxin repressor (DtxR) was first overexpressed in *C. glutamicum* with *hemA*^*M*^ and *hemL* genes, and its combinatorial expression was improved by using effective genetic tools. This engineered strain biosynthesized 68.31 ± 2.15 mg/l of Zn-CP III. Finally, fed-batch fermentation allowed for the production of 132.09 mg/l of Zn-CP III. This titer represents the highest in bacterial production of Zn-CP III reported to date, to our knowledge. This study demonstrates that engineered *C. glutamicum* can be a robust biotechnological model for the production of photosensitizer Zn-porphyrin.

## Introduction

Porphyrins functioning as photo-electrochemical materials are of particular interest in a range of areas related to solar cell technology, battery chemistry, solid/liquid interfaces and biomedical research because of their functional and structural properties that incorporate a metal ion in the center and have great absorption bands in the visible spectrum^1–7^. In particular, Zn-porphyrin derivatives have recently attracted much attention for their great potential application as a promising raw material for sensitizing an organic solar cell in a photovoltaic system that harvests light to convert solar energy into electricity, or in catalyzing multistep reactions at interface^3,4,8–13^. In addition to electronic and physicochemical applications, Zn-porphyrins have been studied as photosensitizers for their antitumor and antibacterial activity in photodynamic therapy-mediated biomedical applications^14^. Furthermore, iron-porphyrin has been used to synthesize bio-based magneto-electric nanocrystals by the heme polymerase enzyme complex and demonstrated as a monomer for advanced nanomaterial synthesis^15^. Most porphyrins used in applications are commonly synthesized from chemical reactions, which have also made great efforts for the high yieldable, cost-efficient and eco-friendly process^16–18^.

Bio-based production of a useful compound is an attractive and alternative route to be able to yield high production levels through a microbial cell factory using a renewable, cost-efficient and eco-friendly source such as glucose. The biological synthesis for important biochemical compounds has recently attracted much attention in various scientific fields such as biotechnology, biomedical, chemical engineering and environmental science^19–22^. However, biosynthesis researches for most porphyrins were rarely reported until recently. In living organisms, porphyrins are commonly biosynthesized through the long and complex heme biosynthesis pathway regulated by eight to nine enzymes. This biosynthesis is initiated from the enzymatic condensation of 5-aminolevulinic acid (ALA) as a common precursor to yield porphobilinogen (PBG). After that, coproporphyrin III (CP III) or protoporphyrin (PP IX), acting as a chassis for various metallated porphyrin derivatives, is biosynthesized from PBG by five or six enzymatic reactions^23^. Metallated porphyrins have various biological and physiological functions. Heme functions as an enzymatic cofactor and electron transfer, chlorophyll is used in photosynthesis, and CP III is an element carrier via chelation of certain metal ions^24,25^.

*Corynebacterium glutamicum*, a gram-positive, nonpathogenic and facultative anaerobic organism, has been frequently used and developed for large-scale production of a variety of amino acids such as l-glutamate and l-lysine, proteins, and monomers for various chemical compounds from renewable biomass^26–31^. Recently, we reported that the engineered *C. glutamicum* produced ALA, a precursor in the heme biosynthesis pathway^32,33^. ALA biosynthesis in almost all bacteria containing *Escherichia coli* and *C. glutamicum* is the rate-limiting step in the heme biosynthesis pathway^34–36^. Hence, ALA biosynthesis has been overcome by two different approaches: 1) engineering of the C5 pathway initiated from l-glutamate or 2) engineering of the C4 pathway initiated from succinyl-CoA with glycine^34–36^. The C5 pathway is processed by three enzymes including glutamyl-tRNA synthetase (encoded by the *gltX* gene) producing l-glutamyl-tRNA from l-glutamate, glutamyl-tRNA reductase (encoded by the *hemA* gene) reducing l-glutamyl-tRNA to glutamate-1-semialdehyde, and glutamate-1-semialdehyde aminotransferase (encoded by the *hemL* gene) biosynthesizing ALA from glutamate-1-semialdehyde by transamination^25^. The C4 pathway is processed by the ALA synthase (encoded by the *alaS* gene) biosynthesizing ALA from succinyl-CoA with glycine^25^. Unlike studies about ALA biosynthesis, as mentioned above, there are only a few studies on the biosynthesis of a porphyrin in bacteria^37–39^. Porphyrin overproduction containing heme, PP IX and/or CP III has been reported in metabolically engineered *E. coli* via overexpression of a number of genes related to the heme biosynthesis pathway through several vectors^37,38^. In the case of *C. glutamicum*, porphyrin production has only been reported as an iron additive for heme production by expressing ALA synthase^39^. In particular, study on the biosynthesis of Zn-porphyrin has been rarely reported, and none reported Zn-porphyrin production in a genetically engineered strain^24,40^.

The heme biosynthesis pathway is long, complicated, and tightly regulated by various factors such as feedback inhibition by the final product and repression of gene expression by the transcriptional regulator^23,25,41–43^. In recent years, transcriptional regulation related to iron and heme homeostasis have been studied in *C. glutamicum*^43–47^. It was revealed that the global transcriptional regulator called diphtheria toxin repressor (DtxR) controlled the expression of genes encoding a variety of membrane protein and transcriptional regulators involved in iron storage, uptake and utilization. In particular, it has been confirmed that heme and iron metabolism are closely involved with each other for homeostasis and are tightly regulated by DtxR protein under conditions dependent on iron concentration^43,45,47^. In addition, it has revealed that the heme responsive regulator activator HrrA (encoded by the *hrrA* gene) repressed the transcription of partial genes included in heme operon^43^. Indeed, when the *hrrA* gene, repressed by the DtxR protein, was deleted from the genome of *C. glutamicum*, the mRNA expression level of partial heme biosynthesis-related genes was upregulated^43^. However, the cell growth of the *hrrA* gene deletion strain was significantly decreased. The direct impact of the DtxR protein for porphyrin production on the control of heme metabolism has not been studied^43^.

**Figure 1:**
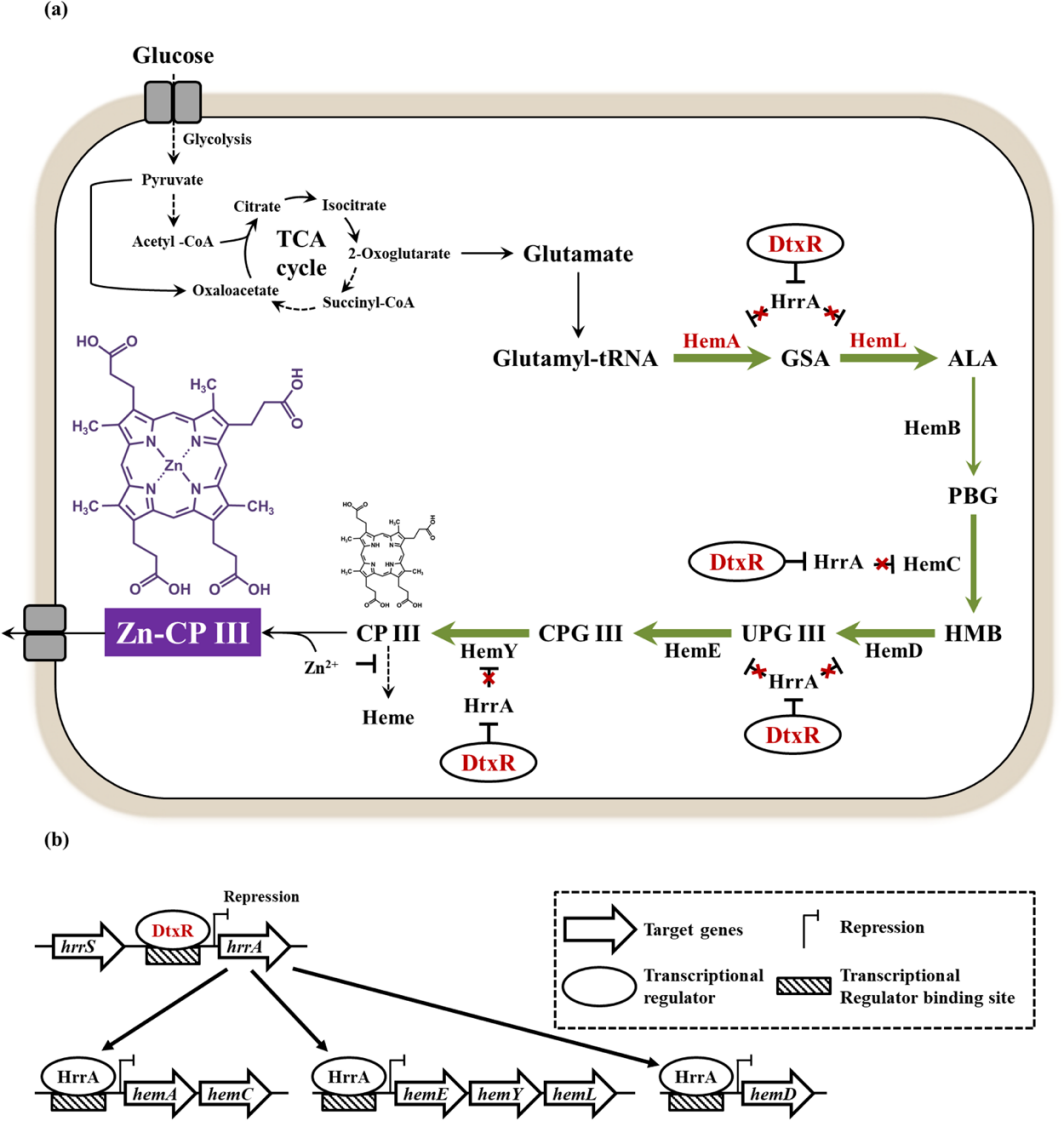
A schematic overview describing the target pathway and overall metabolic strategies for Zn-porphyrin production in *C. glutamicum*. (**a**) The heme biosynthesis pathway used in this study. Glucose indicates a major sole energy source used in this study for cell growth and porphyrin production. Glycolysis starting from glucose is a chain of reactions to form pyruvate, which enters tricarboxylic acid (TCA) cycle with acetyl-CoA. TCA cycle is a series of biochemical reactions providing essential biochemical intermediates, such as 2-oxoglutarate, succinyl-CoA, etc., for cellular energy production and amino acid biosynthesis containing l-glutamate and l-glycine. Zinc-porphyrin biosynthesis is initiated from l-glutamate. The red letter represents the gene overexpression in *C. glutamicum*. The T-shape and red X-shape denote the gene repression by the transcriptional regulator and inactivation of the HrrA protein by gene repression by the DtxR protein, respectively. The solid-line arrow and dashed-line arrow indicate the general metabolic pathway and abbreviated metabolic pathway, respectively. The green arrow denotes enhanced metabolic flux by the combinatorial overexpression of *hemA*^*M*^, *hemL* and *dtxR* genes. The width of each green arrow is proportional to the result of the mRNA expression level of heme biosynthesis genes when the DtxR protein was overexpressed with HemA^M^ and HemL protein. Purple and black colored chemicals are the target product Zn-CP III and porphyrin chassis CP III, respectively. (**b**) The regulatory mechanism of the transcriptional regulator DtxR in *C. glutamicum*. The DtxR protein and HrrA protein bind in the promoter region in front of target genes, respectively, repress the gene expression of those^43,45,47^. Major symbols were described in the dashed black line box. The black line arrow shows processing of regulatory mechanisms of the HrrA protein. Abbreviations: GSA, l-glutamate 1-semialdehyde; ALA, 5-aminolevulinic acid; PBG, porphobilinogen; HMB, hydroxymethylbilane; UPG III, uroporphyrinogen III; CPG III, coproporphyrinogen III; CP III, coproporphyrin III; Zn-CP III, zinc-coproporphyrin III; UP III, uroporphyrin III; HemA, glutamyl-tRNA reductase; *HemL*, glutamate-1-semialdehyde 2,1-aminomutase; HemB, porphobilinogen synthase; HemC, hydroxymethylbilane synthase; HemD, uroporphyrinogen-III synthase; HemE, uroporphyrinogen decarboxylase; HemY, coproporphyrinogen III oxidase; DtxR, diphtheria toxin repressor; HrrA, heme responsive regulator activator.

In this study, we describe the establishment of the *C. glutamicum* platform strain overproducing Zn-CP III and other porphyrins using metabolic engineering for the first time, to our knowledge. We selected *C. glutamicum* as a host strain for Zn-porphyrin production in this study, because this strain overproduces l-glutamate as a starting material in the heme biosynthesis pathway and is a well-developed ALA producer in our previous study^32^. Metabolic engineering for the increased production of Zn-CP III requires enhanced metabolic flux from various metabolites toward coproporphyrin III. Strategies for the enhancement of Zn-CP III production were designed to activate and upregulate the heme biosynthesis pathway and are pictured in Fig. 1a and Supplementary Fig. S1. The strategy for activating the heme biosynthesis pathway is the overexpression of *hemA*^*M*^ (mutated *hemA*) and *hemL* and is reinforced by expressing two genes under the control of a strong promoter. Following this strategy, to achieve the global upregulation of heme operons (Fig. 1b), we first overexpressed DtxR protein into *C. glutamicum* with HemA^M^ and HemL proteins. The combinatorial expression of target genes by effective genetic tools achieved the significant decrease of mRNA expression of the *hrrA* gene, the substantial increase of mRNA expression of heme biosynthesis-related genes, and the highest production of Zn-CP III over engineered strains. Finally, we successfully achieved the biosynthesis of Zn-CP III in fed-batch fermentation supplemented with a zinc source. This study demonstrates that engineered *C. glutamicum* has potential industrial applications as a platform for Zn-porphyrin production.

## Results

### Enhanced activation of the heme biosynthesis pathway by the expression of *hemA*^*M*^ and *hemL* genes under the strong *tac* promoter

**Figure 2:**
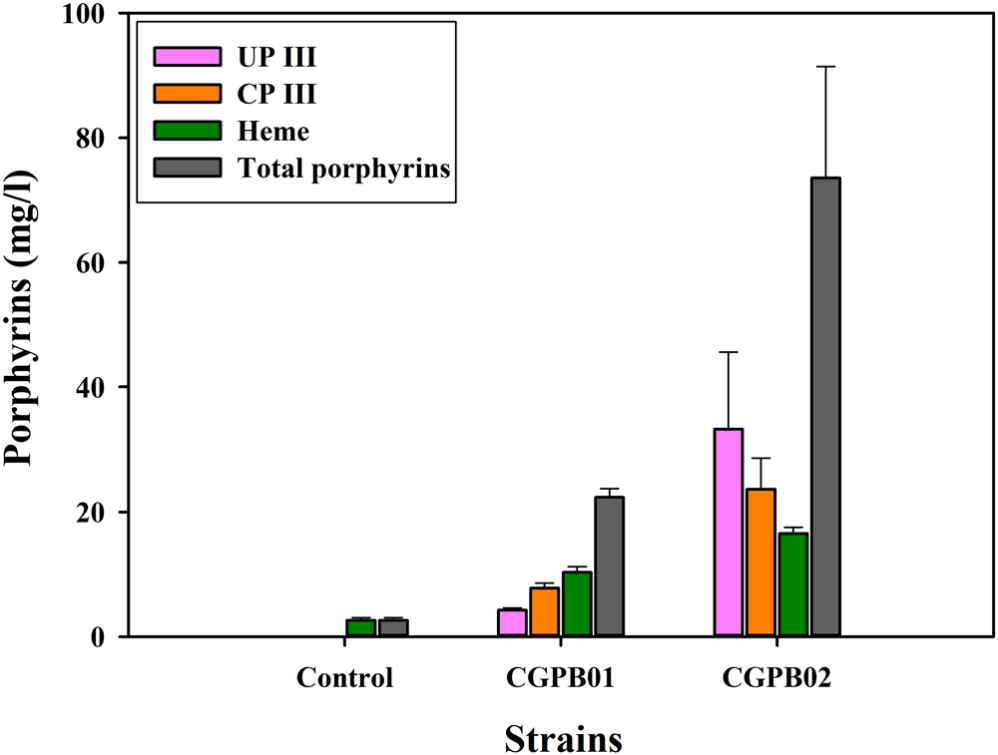
Porphyrin production characteristics of the control, CGPB01 and CGPB02 strains after 72 h of shake-flask cultivation. The x-axis and y-axis represent engineered strains and the titer of porphyrin produced, respectively. The cultivation of all engineered strains was conducted in triplicate, and all data and error bars denote mean values of three independent experiments and standard deviations of mean values, respectively.

ALA production is an important first step for enhanced production of porphyrin derivatives because almost all porphyrins are commonly biosynthesized in the heme biosynthesis pathway using eight molecules of ALA as the precursor^25^. We previously confirmed the potential of engineered *C. glutamicum* as an ALA-producing strain that coexpressed mutated *hemA* (derived from *Salmonella typhimurium*) and *hemL* (derived from *E. coli*) genes by the expression vector pMT-*trc* containing the *trc*-derived promoter and pCG1 origin of replication, named the pHemAL vector(pMT-*trc*::*hemA*^*M*^*L* throughout this article)^32^. In this study, to investigate its potential as a strain for the production of ALA and porphyrins, the engineered strain harboring the pMT-*trc*::*hemA*^*M*^*L* vector was tested. Furthermore, for strong expression of target genes, *hemA*^*M*^ and *hemL* genes were cloned in the other expression vector pMT-*tac* replacing *trc*-derived promoter with the strong *tac* promoter (Supplementary Fig. S1). To analyze metabolites produced, the control strain harboring the empty vector and engineered *C. glutamicum* strains harboring the pMT-*trc*::*hemA*^*M*^*L* and pMT-*tac*::*hemA*^*M*^*L* vectors (named CGPB01 and CGPB02 throughout this article, respectively) were cultivated in modified CGXII medium, adding penicillin G at 12 h for l-glutamate overproduction. Compared with the CGPB01 strain, which produced 338.85 ± 5.44 mg/l of ALA, the CGPB02 strain showed a 1.77-fold increase in the ALA production, corresponding to 600.79 ± 62.47 mg/l of ALA after 72 h of cultivation (Supplementary Fig. S2). Furthermore, while the control strain showed no production of any porphyrins except heme, the CGPB02 strain produced 33.32 ± 12.26 mg/l of uroporphyrin III (UP III), 23.68 ± 4.98 mg/l of CP III, and 16.56 ± 0.98 mg/l of heme, which was 7.79-fold, 3.04-fold, and 1.61-fold more than the CGPB01 strain, respectively (Fig. 2). Additionally, 22.38 ± 1.37 and 73.56 ± 17.89 mg/l of total porphyrins were produced in CGPB01 and CGPB02, respectively. Maximum cell growth was not affected by the expression of *hemA*^*M*^ and *hemL* genes under the *tac* promoter (Supplementary Fig. S2). This result indicates that the expression of *hemA*^*M*^ and *hemL* genes is important for activation of the heme biosynthesis pathway in *C. glutamicum*, and the expression under the *tac* promoter was strong enough to increase the metabolic flux of the heme biosynthesis pathway compared with the *trc*-derived promoter.

### Enhanced metabolic flux toward a porphyrin chassis CP III by the combinatorial expression of the *hemA*^*M*^*, hemL and dtxR* genes

**Figure 3:**
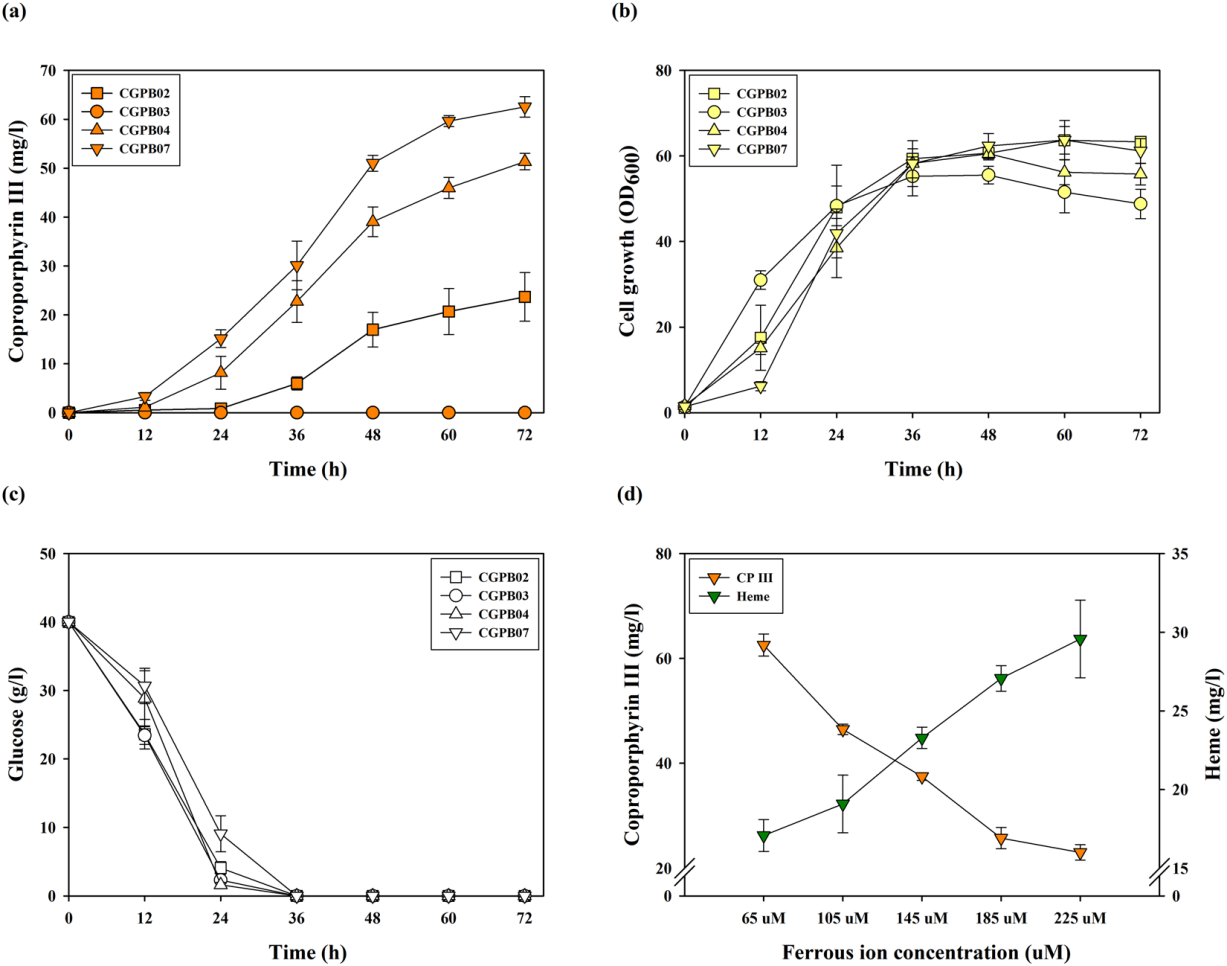
Production and growth characteristics of the CGPB02, CGPB03, CGPB04, and CGPB07 strains during 72 h of shake-flask cultivation. (**a**) CP III production in engineered strains. The x-axis and y-axis represent time and the titer of CP III produced, respectively. (**b**) Cell growth of engineered strains. The x-axis and y-axis denote time and OD_600_, respectively. (**c**) Glucose consumption of engineered strains. The x-axis and y-axis indicate time and glucose concentration in the medium, respectively. (**d**) CP III and heme production in the CGPB07 strain in the culture condition of various iron sulfate concentrations. The x-axis, left y-axis and right y-axis represent the iron sulfate concentration and titer of CP III and heme produced, respectively. The cultivation of all engineered strains was conducted in triplicate, and all data and error bars denote mean values of three independent experiments and standard deviations of mean values, respectively. Symbols: square, CGPB02 strain; circle, CGPB03 strain; triangle, CGPB04 strain; inverted triangle, CGPB07 strain; orange color, CP III production; green color, heme production; yellow color, cell growth; white color, glucose.

In this study, we selected CP III as a final chassis for Zn-porphyrin biosynthesis. It has been revealed that not only CP III acted as a chelate of zinc, but also Zn-CP III was biosynthesized from CP III produced in bacteria including wild type *Paracoccus denitrificans* and *Streptomyces* sp. in culture medium supplemented with zinc sulfate^24,40^. As mentioned above, the transcription regulation mechanism between partial heme operon and the HrrA protein in *C. glutamicum* has been studied, although the cell growth of the *hrrA* gene deletion strain was significantly decreased^43,45^. Hence, we hypothesized that the DtxR protein may serve as an effective enhancer of the heme biosynthesis pathway. To verify this hypothesis, the *dtxR* gene (derived from *C. glutamicum*) encoding the DtxR protein was introduced into the control strain and the CGPB02 strain, named the CGPB03 and CGPB04 strains, respectively. Contrary to expectations, while the production of CP III and UP III in the CGPB03 strain was not detected, the CGPB04 strain showed a 1.82-fold and 2.17-fold increase (60.90 ± 3.26 and 51.35 ± 1.69 mg/l) of UP III and CP III production over the CGPB02 strain, respectively (Fig. 3a, Supplementary Fig. S3). Although the overexpression of the *hemA*^*M*^ and *hemL* genes somewhat delayed the exponential growth phase, whether or not the *dtxR* gene was present, the maximum cell growth and glucose consumption were not affected (Fig. 3b,c). These results show that the combinatorial expression of *dtxR* with *hemA*^*M*^ and *hemL* not only overcomes the rate-limiting step but also improves CP III production by enhancing the metabolic flux of the heme biosynthesis pathway without the critical negative effect of cell growth.

To enhance the metabolic flux of the heme biosynthesis pathway toward CP III, the *hemA*^*M*^, *hemL* and *dtxR* genes were additionally cloned in an *E. coli*-*C. glutamicum* shuttle vector pEKEx2 containing the *tac* promoter and pBL1 origin of replication. A pBL1 origin of replication contained in Shuttle vector pEKEx2 shows 8–30 copy numbers per cell, and a pCG1 origin of replication contained in pMT-*tac* shows 13–22 copy numbers per cell^48,49^. Expression of the *hemA*^*M*^*, hemL* and *dtxR* genes using the pEKEx2 vector was more effective at producing CP III than using the pMT-*tac* vector. Compared with the CGPB04 strain, the engineered strain harboring pEKEx2::*hemA*^*M*^*LdtxR* (named the CGPB07 strain herein) showed a 1.22-fold increase in CP III production, corresponding to 62.54 ± 2.09 mg/l (Fig. 3a). Interestingly, UP III production in the CGPB07 strain was decreased 1.55-fold compared to the CGPB04 strain (Supplementary Fig. S3). The result means that the metabolic flux from UP III to CP III may be extended by the reinforced expression of *hemA*^*M*^*, hemL* and *dtxR* genes through the pEKEx2 vector. Therefore, the CGPB07 strain is more useful for Zn-CP III production compared to other engineered strains.

The DtxR protein uses two ferrous iron as an essential cofactor^45^. Hence, cultivation with various concentrations of iron was performed on the CGPB04 and CGPB07 strains for enhanced production of CP III. However, along with increasing the iron concentration, CP III production in both the CGPB04 and CGPB07 strains decreased constantly while heme production was increased (Fig. 3d, Supplementary Fig. S4). These results mean that ferrous iron added in the culture medium may be used in processes to biosynthesize heme from CP III.

### Global upregulation of the heme biosynthesis pathway by the transcriptional regulator DtxR

**Figure 4:**
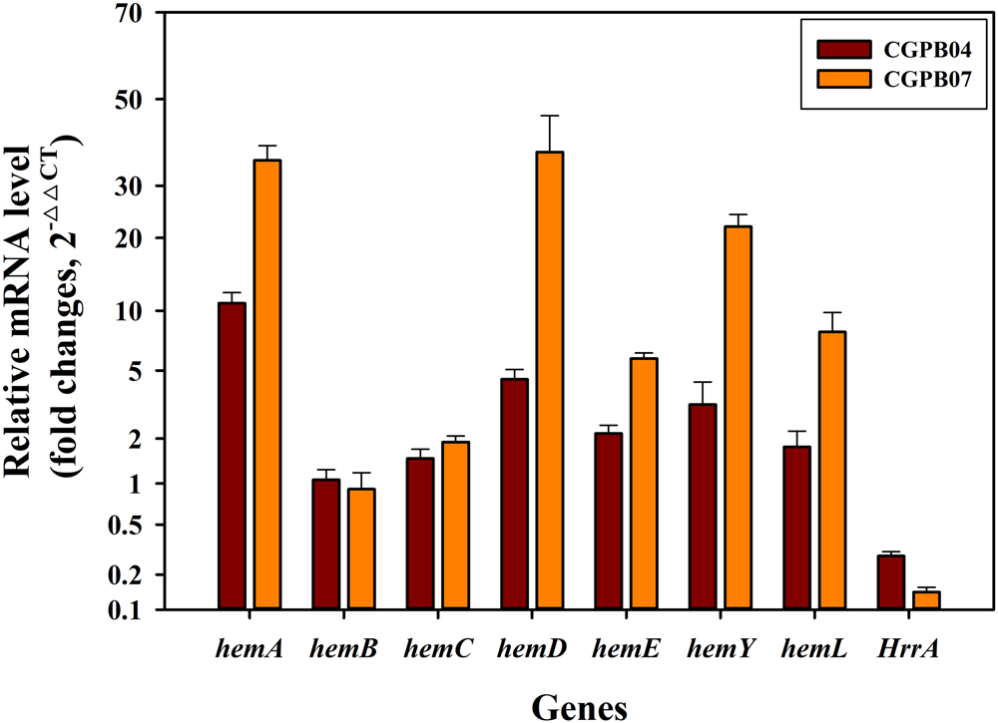
The analysis of mRNA expression levels of porphyrin biosynthesis related-genes in the CGPB02, CGPB04 and CGPB07 strains by qRT-PCR. To analyze mRNA expression levels of target genes, all engineered strains were incubated in CGXII medium with 4% glucose for 12 h. Relative mRNA expression levels of *hemA*, *hemL*, *hemB, hemC, hemD, hemE, hemY*, and *hrrA*, measured in the CGPB04 and CGPB07 strains, were compared with those of the CGPB02 strain. The 16 S rRNA level in all engineered strains was used as an internal reference for normalization. The mRNA analysis of all engineered strains was conducted in triplicate, and all data and error bars represent mean values of three independent experiments and standard deviations of mean values, respectively.

It has been reported that the mRNA expression level of *hemA, hemC* (encoding porphobilinogen deaminase)*, hemE* (encoding uroporphyrinogen decarboxylase)*, hemY* (encoding protoporphyrinogen oxidase) and *hemL* was decreased when the *hrrA* gene was deleted in *C. glutamicum*^43^. However, transcriptional analysis of *hemB* and *hemD* (encoding uroporphyrinogen-III synthase) genes in the *hrrA* gene deletion strain as well as the transcriptional regulation of the heme biosynthesis pathway by the overexpression of the DtxR protein has not been studied yet. In particular, the DtxR protein has never been used for porphyrins production, to our knowledge. To investigate the positive effect of the DtxR protein in enhancing the metabolic flux of the heme biosynthesis pathway, we analyzed the relative mRNA expression of heme biosynthesis-related genes when the DtxR protein was overexpressed. Compared to the CGPB02 strain, the mRNA expression levels of the *hemA*, *hemL*, *hemC*, *hemD*, *hemE* and *hemY* genes in the genome directly involved in CP III production after the precursor synthesis were all increased in the CGPB04 strain, which showed 10.83 ± 1.21-, 1.77 ± 0.46-, 1.48 ± 0.22-, 4.50 ± 0.55-, 2.15 ± 0.27- and 3.23 ± 1.19-fold increases respectively (Fig. 4). Furthermore, the mRNA expression level of the *hrrA* gene in the CGPB04 strain was significantly decreased 0.28 ± 0.02-fold over the CGPB02 strain. However, the *hemB* mRNA expression level did not change, and this result means that the *hemB* gene may not be regulated by the HrrA protein. Taken together, these data indicate that genes related to the heme biosynthesis pathway were mostly upregulated by *hrrA* gene repression via overexpression of the DtxR protein, which may result in increasing the total porphyrin production in the engineered strain. In addition, the mRNA expression levels of the *hemA, hemL, hemC, hemD, hemE* and *hemY* genes in the CGPB07 strain were more increased than those of the CGPB04 strain, and showed the 35.55 ± 3.37-, 1.89 ± 0.18-, 37.40 ± 8.58-, 5.79 ± 0.41-, 22.02 ± 2.20- and 7.92 ± 1.88-fold increase over the CGPB02 strain, respectively. The *hrrA* mRNA expression level in the CGPB07 strain was more decreased than that of the CGPB04 strain, showed the 0.14 ± 0.01-fold decrease over the CGPB02 strain. The result means that the mRNA expression of heme biosynthesis-related genes may be improved by the *hrrA* gene repression by the improved expression of the DtxR protein through the pEKEx2 vector.

### Zn-CP III biosynthesis by engineered *C. glutamicum* strains by the combinatorial expression of the *hemA*^*M*^*, hemL and dtxR* genes

**Figure 5:**
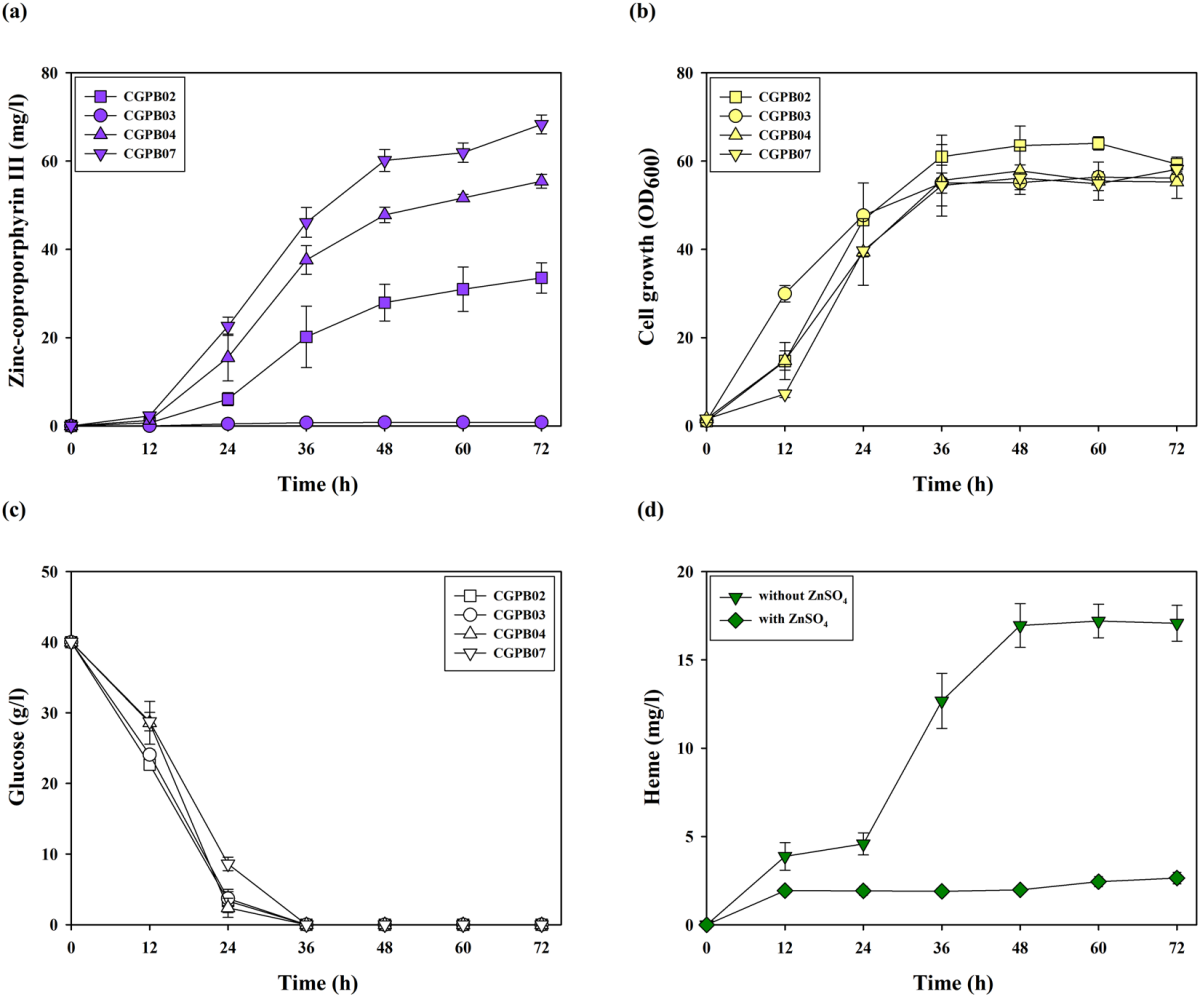
Production and growth characteristics of the CGPB02, CGPB03, CGPB04, and CGPB07 strains during 72 h of shake-flask cultivation supplemented with zinc sulfate. (**a**) Zn-CP III production in engineered strains. The x-axis and y-axis represent time and the titer of Zn-CP III produced, respectively. (**b**) Cell growth of engineered strains. The x-axis and y-axis denote time and OD_600_, respectively. (**c**) Glucose consumption of engineered strains. The x-axis and y-axis indicate time and glucose concentration in the medium, respectively. (**d**) Comparison of heme production in the culture with and without zinc sulfate in the CGPB07 strain. The x-axis and y-axis represent time and titer of heme produced, respectively. The cultivation of all engineered strains was conducted in triplicate, and all data and error bars denote mean values of three independent experiments and standard deviations of mean values, respectively. Symbols: square, CGPB02 strain; triangle, CGPB04 strain; inverted triangle or diamond (in result supplemented with zinc sulfate), CGPB07 strain; purple color, Zn-CP III production; green color, heme production; yellow color, cell growth; white color, glucose.

For Zn-CP III production, CP III-producing engineered strains were shake-cultivated in a glucose-minimal medium supplemented with zinc sulfate for 72 h, and most engineered strains except for the CGPB03 strain successfully produced Zn-CP III (Fig. 5a). Compared with the CGPB02 strain that produced 33.54 ± 3.44 mg/l of Zn-CP III, the CGPB04 and CGPB07 strains showed 1.65- and 2.04-fold increases in Zn-CP III production, corresponding to 55.44 ± 1.58 and 68.31 ± 2.15 mg/l, respectively. Similarly, as in the case of CP III, the CGBP07 strain showed the highest titer of Zn-CP III in bacteria reported to date, and the growth pattern and glucose consumption of all engineered strains were rarely affected (Fig. 5b,c).

Interestingly, relative to the total titer of CP III produced in the CGPB07 strain cultured without zinc sulfate, the total titer of Zn-CP III produced in the strain cultured with zinc sulfate was increased to 68.31 ± 2.15 mg/l from 62.54 ± 2.09 mg/l of CP III without certain genetic modifications, corresponding to a further 4.4% increase (Figs 3a, 5a). On the other hand, the total titer of heme produced in the strain cultured with zinc sulfate was decreased compared to that produced in the strain cultured without zinc sulfate, from 17.08 ± 0.10 mg/l to 2.64 ± 0.32 mg/l (Fig. 5d). This result means that CP III was partially used in Zn-CP III production rather than in heme production. The absorption spectrum of metabolites produced in the CGPB07 strain when zinc sulfate was supplemented showed not only the strongest feature at 406 nm but also minor features at 538 and 574 nm, representing optical characteristics of Zn-CP III (Supplementary Fig. S5) similar to that of previous studies^24,40^. Typically, CP III has a maximum Soret band at 393 nm, consistent with the absorption maximum of metabolites produced in the strain cultured without zinc sulfate. These results suggest that engineered *C. glutamicum* strains successfully produced Zn-CP III, and efficiently used robust porphyrin metabolism to biosynthesize Zn-CP III.

### Fed-batch fermentation for enhanced biosynthesis of Zn-CP III in engineered *C. glutamicum*

**Figure 6:**
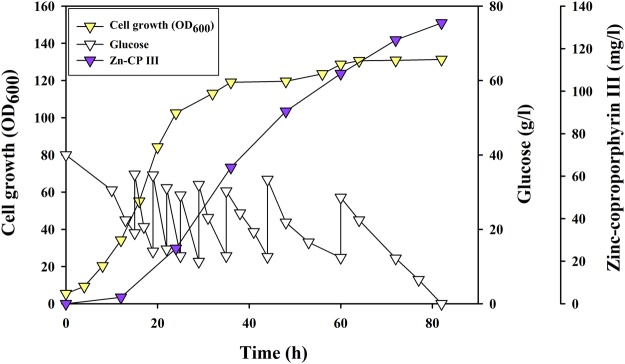
The fed-batch fermentation profile of the CGPB07 strain for Zn-CP III production. The x-axis, left y-axis, right y-axis, and right y-offset denote time, OD_600_, glucose, and Zn-CP III produced, respectively. Symbols: inverted triangle, CGPB07 strain; purple color, Zn-CP III production; yellow color, cell growth; white color, glucose.

To test the potential of the CGPB07 strain for large-scale production of Zn-CP III, we carried out 1.8- l fed-batch fermentation in a 5 l fermenter (Fig. 6). Glucose feeding was initiated after 15 h of fermentation and was additionally injected whenever the glucose concentration was below 20 g/l. During fed-batch fermentation of the CGPB07 strain, the maximum titer reached 132.09 mg/l of Zn-CP III in 82 h of fermentation in which the total input of glucose was 350.5 g. The overall yield and productivity were 1.27 mg/g glucose and 1.61 mg/l/h, respectively. The specific growth rate in the exponential growth phase (between 8 and 20 h) was 0.12/h. The maximum OD_600_ was 131.33, corresponding to the measured cell biomass of 37.04 g/l at the end of fermentation.

## Discussion

Much research has recently advanced Zn-porphyrin and porphyrin derivatives as promising raw material for solar cell devices, solid/liquid interface and photodynamic therapy over the past several years^1–6^. Until now, the development of porphyrin production in bacteria has lagged behind precursor ALA production presumably because of the complex synthetic processes and regulatory mechanisms of the heme biosynthetic pathway. Toriya *et al*. found that wild type *Streptomyces* sp. strain AC8007 biosynthesizes Zn-CP III (10 mg/l), but there was no metabolic design for overproduction of Zn-CP III in this wild type strain^40^. Kwon *et al*. reported successful production of CP III, PP IX and heme (89 ± 9 μmol/l of total porphyrins, corresponding to approximately 50 mg/l of total porphyrins) in *E. coli* by overexpressing eight genes related to heme biosynthesis^37^. However, the introduction of many genes and vectors may require excessive use of antibiotic resistance markers and reduce cell growth by metabolic burden^50^. Indeed, engineered *E. coli* strains, named as the ECPB01, ECPB02 and ECPB03 strains, less produced Zn-CP III than engineered *C. glutamicum* strains, corresponding to 1.93 ± 0.05 mg/l, 1.87 ± 0.07 mg/l and 1.84 ± 0.05 mg/l, respectively (Supplementary Fig. S6). The porphyrin production in the engineered *E. coli* strain overexpressing the HemA^M^ and HemL proteins (C5 pathway) in this study was similar to that in the engineered strain overexpressing the ALA synthase (C4 pathway) used in the study of Kwon *et al*.^37^. Furthermore, the DtxR protein only worked in *C. glutamicum*, but not in *E. coli* (Fig. 3a, Supplementary Figs S3, S6). This result means that *C. glutamicum* is suitable for Zn-CP III and have the possibility of performing the further metabolic engineering. In the field of microbial engineering, pertinent gene expression and regulation of the target biosynthesis pathway are important factors for high-yield production of a target product^51^. In this study, we confirmed the potential of *C. glutamicum* as a platform strain for Zn-CP III and porphyrin production. The activation and upregulation of the long and complex heme biosynthesis pathway by the transcriptional regulator DtxR alongside HemA^M^ and HemL proteins in *C. glutamicum* can allow for efficient biosynthesis of Zn-CP III. Furthermore, target gene expression by the efficient genetic tool such as replacement of the strong promoter and origin of replication achieved the high production of CP III, Zn-CP III and Heme. Furthermore, the mRNA expression of most heme biosynthesis genes except for the *hemB* gene was significantly improved in the CGPB07 strain (Fig. 4). Interestingly, while the *hemD* gene, which is located at 500-bp upstream of the *hemB* gene, was upregulated by the *hrrA* gene repression, the *hemB* gene expression was not affected in both the CGPB04 and CGPB07 strains. Even though the *hemB* gene is close to the *hemD* gene in genome, their expression may be regulated by different expression factors containing a promoter and repressor because it is predicted that they do not exist as an operon (an operon was predicted at http://www.coryneregnet.de/)^43^. Based on these results, It can be speculated that *hemB* and *hemD* genes are individually expressed by different factors and that there exists no binding site for the HrrA protein in the promoter region of the *hemB* gene. Frunzke *et al*. has identified that the HrrA protein bound in the promoter region of *hemA* and *hemE* genes although the promoter region of *hemD* and *hemB* genes remained unclear^43^. Nonetheless, the enhanced mRNA expression level of most heme biosynthesis genes by the combinatorial expression of *hemA*^*M*^, *hemL* and *dtxR* genes induced substantial porphyrin production (Figs 3a, 4, Supplementary Fig. S3). Interestingly, in the CGPB03 strain overexpressing the *dtxR* gene alone, most of the porphyrins except for heme were not produced (Supplementary Fig. S3). We suggest the reason may be because the native HemA protein overexpressed by the DtxR protein is feedback-inhibited by the final product heme^41–43^.

In terms of the insertion of a zinc ion into metal-free porphyrin, CP III may be more suitable for Zn-porphyrin synthesis than PP IX because CP III has the characteristic of spontaneous chelation of a zinc ion without any energy requirement while PP IX requires an enzyme reaction for Zn-PP IX biosynthesis^24,52,53^. Indeed, as described in our results regarding CP III, Zn-CP III and heme concentration, CP III generated for subsequent biosynthesis pathways was used for Zn-CP III production instead of heme production (Figs 3, 5). Dailey *et al*. recently revealed that the Actinobacteria and Firmicutes uses CP III instead of PP IX in heme biosynthesis^54^. *C. glutamicum*, which belongs to phylum Actinobacteria, is able to biosynthesize heme from a coproporphyrin-dependent pathway. As reported in Fig. 3, CP III accumulation was shown in metabolites produced in the CGPB02, CGPB04, and CGPB07 strains. However, none of the engineered strains constructed in this study produced PP IX despite increased mRNA expression levels of the *hemN* gene (encoding coproporphyrinogen III oxidase) (Supplementary Fig. S7). Furthermore, we confirmed that PP IX was produced when coproporphyrinogen III oxidase derived from *E. coli* with *hemA*^*M*^*, hemL* and *dtxR* genes was heterogeneously expressed in *C. glutamicum* (Supplementary Fig. S8). This observation suggests that *C. glutamicum* may not have a protoporphyrin-dependent pathway for heme biosynthesis, and therefore is suitable in terms of being a coproporphyrin-producing platform strain.

Even though the mRNA expression levels of the *hemA, hemL, hemB, hemC, hemD, hemE* and *hemY* genes for CP III production, as well as that of the *hemH* gene, were increased, heme production in the CGPB04 strain was slightly decreased, compared with the CGPB02 strain (Supplementary Figs S3, S7). Regarding this result, we speculate that the DtxR protein and heme competed for ferrous ion as a cofactor and required metal ions, respectively. Although the heme production from CP III was increased in the culture supplemented with iron sulfate of the CGPB07 strain, the bioconversion yield from CP III to heme was still low at 31.55 ± 3.39% (Fig. 3d). It is tempting to speculate that the final biosynthetic section from CP III to heme may be a major bottleneck for heme biosynthesis. Kwon *et al*. suggested that the limited transport of a metal ion into the cell and the low activity of the HemH protein *in vivo* may limit heme biosynthesis in the recombinant *E. coli*^37^. It has been recently reported that heme biosynthesis in the coproporphyrin-dependent pathway is performed by the HemQ protein which decarboxylates coproheme resulting in heme^54–57^. Hence, a mechanistic and enzymatic perspective of the coproporphyrin-dependent pathway in *C. glutamicum* is required for enhanced heme production. Nevertheless, our result regarding heme concentration in the CGPB07 strain showed the high-level production, corresponding to 29.57 ± 2.46 mg/l (Fig. 3d).

In summary, we demonstrated the possibility of *C. glutamicum* as a platform strain for the microbial production of Zn-porphyrin Zn-CP III as well as heme, and the titer of the porphyrins in the final engineered strain CGPB07 are the highest, reported to date in bacteria (Figs 3d, 6, Supplementary Fig. S3). These bio-based compounds can be used as a valuable material in various research related to solar cell photosensitizers, biological sensors as well as biomedical application such as antimicrobial, antitumor and anticancer photodynamic therapy^1–15,58,59^. In further study, large scale production of Zn-porphyrin in bacteria by microbial and metabolic engineering can be directly applied to solar cell technology and biomedical research. Indeed, Toriya *et al*. has directly applied Zn-CP III biosynthesized from wild type *Streptomyces* sp. used in their previous study to photodynamic therapy for antitumor effect in mice^40,59^. To enhance porphyrin biosynthesis and increase the diversity of types of porphyrins, further microbial engineering in *C. glutamicum* should be performed, including rational pathway engineering of the endogenous metabolism toward increased availability of an additional rate-limiting step, fine-tuning of enzyme engineering for improving target enzymes representing low activity, and the development of transport systems for efficient substrate supply.

## Materials and Methods

### Reagents and chemicals

Cell culture media used in this study was purchased from Daejung chemical Co. (Korea) and BD Bioscience (USA). Genomic DNA and plasmid DNA were prepared using Wizard® Genomic DNA Purification Kit (Promega, USA) and GeneAll® Exprep Plasmid Kit (GeneAll, Korea), respectively. Oligo synthesis and DNA sequencing were performed by Macrogen (Korea) and Cosmogenetech (Korea), respectively. Restriction enzymes, Taq DNA polymerase and T4 DNA ligase were purchased from New England Biolabs (USA), GeneAll (Korea) and Promega (USA), respectively. DNA fragments treated by either polymerase, ligase or restriction enzymes were purified by GeneAll® Expin™ Gel SV Kit (GeneAll, Korea). HPLC-grade chemicals such as acetonitrile and methanol were purchased from Duksan (Korea). 5-Aminolevulinic acid hydrochloride (Sigma-Aldrich, USA), Uroporphyrin III dihydrochloride (Echelon Biosciences, USA), coproporphyrin III dihydrochloride (Santa Cruz biotechnology, USA), protoporphyrin IX (Sigma-Aldrich) and heme (Sigma-Aldrich, USA) were used as standard chemicals for the quantitative analysis. Zn-CP III was prepared by the modified method described by Anttila *et al*.^24^. For quantitative real time-PCR (qRT-PCR) analysis, Hybrid-R prep kit, M-MLV reverse transcriptase and HiPi Real-Time PCR 2x Master Mix (SYBR green) were purchased from GeneAll (Korea), Bioneer (Korea) and Elpisbio (Korea), respectively.

### Strains and plasmids

All plasmids and bacterial strains constructed and used in this study are listed in Table 1. *E. coli* DH5α was chosen as the cloning host strain, and *C. glutamicum* ATCC 13826 was used in the construction of recombinant strains and experiments for porphyrin production in this study. *S. typhimurium*, *E. coli* and *C. glutamicum* strains were used to obtain *hemA*, *hemL* and *dtxR* genes from genomic DNA, respectively.

**Table 1:** Bacterial strains and plasmids used in this study.

Strain or plasmid	Genotype or construct	Reference or source
**Strains**		
*E. coli*		
DH5α	F^−^, *deoR*, *endA1*, *gyrA96*, *hsdR17*(rk^−^mk^+^), *recA1*, *relA1*, *supE44*, *thi-1*, Δ(*lacZYA- argF*)*U169*, (Phi80*lacZ*delM15)	Invitrogen^a^
BL21 (DE3)	F^−^ *ompT gal dcm lon hsdS*_*B*_ (r_B_^−^ m_B_^−^) λ(DE3 [*lacI lacUV5-T7* gene 1 *ind1 sam7 nin5*])	Invitrogen^a^
ECPB01	*E. coli* BL21, pMT-*tac*::*hemA*^*M*^*L*	This study
ECPB02	*E. coli* BL21, pMT-*tac*::*hemA*^*M*^*LdtxR*	This study
ECPB03	*E. coli* BL21, pEKEx2::*hemA*^*M*^*LdtxR*	This study
*C. glutamicum*		
ATCC 13826	l-Glutamate producing industrial strain	ATCC^b^
Control strain	*C. glutamicum* ATCC 13826, pMT-*trc*	This study
CGPB01	*C. glutamicum* ATCC 13826, pMT-*trc*::*hemA*^*M*^*L*	This study
CGPB02	*C. glutamicum* ATCC 13826, pMT-*tac*::*hemA*^*M*^*L*	This study
CGPB03	*C. glutamicum* ATCC 13826, pMT-*tac*::*dtxR*	This study
CGPB04	*C. glutamicum* ATCC 13826, pMT-*tac*::*hemA*^*M*^*LdtxR*	This study
CGPB05	*C. glutamicum* ATCC 13826, pEKEx2::*hemA*^*M*^*L*	This study
CGPB06	*C. glutamicum* ATCC 13826, pEKEx2::*dtxR*	This study
CGPB07	*C. glutamicum* ATCC 13826, pEKEx2::*hemA*^*M*^*LdtxR*	This study
**Plasmids**		
pMT-*trc*	*C. glutamicum-E. coli* shuttle vector, P_trc_, Amp^R^, Kan^R^, pCG1 ori	Ramzi *et al*. (2015)
pMT-*tac*	*C. glutamicum-E. coli* shuttle vector, P_tac_, Amp^R^, Kan^R^, pCG1 ori	Joo *et al*. (2017)
pMT-*trc*::*hemA*^*M*^*L*	pMT-*trc* carrying *hemA*^*M*^ *and hemL* genes	Ramzi *et al*. (2015)
pMT-*tac*::*dtxR*	pMT-*tac* carrying *dtxR* gene	This study
pMT-*tac*::*hemA*^*M*^*L*	pMT-*tac* carrying *hemA*^*M*^ *and hemL* genes	This study
pMT-*tac*::*hemA*^*M*^*LdtxR*	pMT-*tac* carrying *hemA*^*M*^*, hemL, and dtxR* genes	This study
pEKEx2	*C. glutamicum-E. coli* shuttle vector, P_tac_, lacI, Kan^R^, pBL1 ori	This study
pEKEx2::*dtxR*	pEKEx2 carrying *dtxR* gene	This study
pEKEx2::*hemA*^*M*^*L*	pEKEx2 carrying *hemA*^*M*^ *and hemL* genes	This study
pEKEx2::*hemA*^*M*^*LdtxR*	pEKEx2 carrying *hemA*^*M*^*, hemL, and dtxR* genes	This study

^a^Invitrogen Corporation, Carlsbad, California, USA. ^b^American Type and Culture Collection, Manassas, USA.

All oligonucleotides for the construction of recombinant vectors were listed in Supplementary Table S1. pMT-*tac* and pEKEx2 vectors were used as *E. coli* and *C. glutamicum* shuttle vectors and for the expression of target genes in *C. glutamicum*. To express *hemA*^*M*^ and *hemL* genes under the control of the *tac* promoter, the *hemA*^*M*^ and *hemL* gene operon was amplified with HemA^M^ *Bam*HI F and *HemL Not*I R primers using pMT-*trc*::*hemA*^*M*^*L* vector as a template. The *hemA*^*M*^-*hemL* gene fragment was ligated into *Bam*HI- *Not*I restriction sites of the pMT-*tac* vector by T4 DNA ligation, resulting in the pMT-*tac*::*hemA*^*M*^*L* vector. For the construction of pMT-*tac*::*hemA*^*M*^*LdtxR* and pMT-*tac*::*dtxR* vectors, the *dtxR* gene was amplified with DtxR *Not*I F and DtxR *Not*I R primers using pMT-*tac*::*hemA*^*M*^*L* and pMT-*tac* vectors as a template, respectively. To prevent self-ligation by one-cut restriction digestion, the digested sample was treated with Cloned Alkaline Phosphatase (Takara, Japan). The *dtxR* gene fragment was ligated into the *Not*I restriction sites of pMT-*tac*::*hemA*^*M*^*L* and pMT-*tac* vectors, resulting in pMT-*tac*::*hemA*^*M*^*LdtxR* and pMT-*tac*::*dtxR* vectors. In the same manner, the pEKEx2::*hemAL* vector was constructed by means of restriction digestion and ligation into *Pst*I-*Sal*I sites of the pEKEx2 vector. The *dtxR* gene was also introduced into *Sal*I-*Bam*HI sites of pEKEx2 and pEKEx2::*hemA*^*M*^*L* vectors, resulting in pEKEx2::*dtxR* and pEKEx2::*hemA*^*M*^*LdtxR*. Except for an adjacent gene from a promoter, a ribosomal binding site (AAGGAG) was inserted between downstream of the stop codon and upstream of the start codon in individual genes. Transformation of all recombinant vectors in *E. coli* and *C. glutamicum* was performed by the methods previously described^27^.

### Culture medium and conditions

For the construction of engineered strains, *E. coli* and *C. glutamicum* were grown overnight in shaking cultivation at 37 °C and 30 °C, respectively, and 200 rpm in Luria–Bertani medium. *C. glutamicum* wild type and engineered strains were grown overnight in shaking cultivation at 30 °C and 150 rpm in brain–heart infusion (BHI) –. The seed culture for porphyrin production was washed once with modified CGXII medium and transferred to main flask cultivation at a starting OD_600_ of 1. Main flask cultivation for porphyrin production was conducted in 500 ml baffled flasks containing 100 ml modified CGXII medium containing 40 g/l glucose and 1 mM Isopropyl *β*-d-1-thiogalactopyranoside (IPTG). For glutamate overproduction, 6 U/ml penicillin G was supplemented after 12 h of cultivation. Modified CGXII medium consists of 20 g (NH_4_)_2_SO_4_, 5 g urea, 1 g KH_2_PO_4_, 1 g K_2_HPO_4_, 42 g 3-morpholinopropanesulfonic acid (MOPS), 0.25 g MgSO_4_·7H_2_O, 10 mg CaCl_2_, 10 mg FeSO_4_·7H_2_O, 0.1 mg MnSO_4_·H_2_O, 1 mg ZnSO_4_·7H_2_O, 0.31 mg CuSO_4_·5H_2_O, 0.02 mg NiCl_2_·6H_2_O and 0.2 mg biotin per liter. Recombinant vectors in engineered strains were maintained by relevant concentrations of antibiotics (50 mg/l ampicillin and 25 mg/l kanamycin).

For fed-batch fermentation, stock cells were streaked onto BHI agar plates with 25 mg/l kanamycin and cultured for 30 h of standing incubation at 30 °C. One colony was used to inoculate 20 ml of the first seed culture and grown overnight in shaking cultivation at 30 °C and 150 rpm. The first seed culture was transferred to 200 ml of baffled flasks containing the second seed culture. The second seed culture was washed once with modified CGXII medium and transferred to fermenter containing 1.8 l modified CGXII medium, 40 g/l glucose, 1 mM IPTG and 25 mg/l kanamycin. The fermentation was performed at 30 °C and 300 to 600 rpm. The pH was automatically maintained at 6.8 by 4 M NaOH and 5 M H_2_SO_4_. Air aeration rate and dissolved oxygen were maintained at 1 vvm and 30% of air saturation, respectively. The foam was removed by the addition of 1:10 diluted antifoam Y-30 emulsion (Sigma-Aldrich). When glucose was lower than 20 g/l, 50% glucose stock solution was manually fed.

### Analytical methods

Cell growth of all strains used in this study was measured at OD_600_ using a UV–vis spectrophotometer (Mecasys Co., Ltd., Korea). The dry cell weight was experimentally determined on the basis of the correlation model OD_600_ 1 = 0.282 g dry cell weight (DCW)/l and used for the calculation of biomass concentration of fed-batch fermentation. Glucose concentration was estimated using a Glucose (GO) Assay Kit (Sigma-Aldrich, USA). For measurement of intracellular metabolites, 1 ml of cell pellet was harvested by centrifugation (13,000 rpm at 4 °C for 3 minutes), and the supernatant excluding the cell pellet was used for the measurement of extracellular metabolites. For the extraction of the metabolite from cells, including inter alia, heme and other porphyrins, the cell pellet was disrupted using-modified actone:HCl extraction methods described by Espinas *et al*.^60^. After 1 ml of acetone:HCl (95:5) buffer was added to the cell-harvested tube, the mixture was vortexed and diluted with 1 ml of 1 M NaOH. The intracellular sample which was disrupted and supernatant was filtered using an MCE (Mixed Cellulose Ester) filter (Hyundai micro, Korea) for concentration analysis. Porphyrin concentration was determined using a high-performance liquid chromatography (HPLC) system (Waters Corporation, USA), which consists of a dual λ absorbance detector (Waters 2487), binary HPLC pump (Waters 1525), and autosampler (Waters 2707). The filtered sample was separated in a SUPELCOSIL™ LC-18-DB HPLC Column 5 μm particle size, L × I.D. 250 × 4.6 mm (Supelco Inc., USA) using a linear gradient method of 20–95% solvent A in B at 40 °C. Solvent A is a 10:90 (v/v) HPLC grade methanol:acetonitrile mixture, and solvent B is a 0.5% (v/v) trifluoroacetic acid (TFA) in HPLC grade water. The flow rate was 1 ml/min for 40 minutes, and the absorbance was determined at 400 nm. The data were estimated using Waters Empower-3 software. ALA concentration was measured using the colorimetric assay called Ehrlich’s reagent^61^. One volume of the supernatant was chemically reacted with 0.5 volume of 1 M sodium acetate buffer (pH 4.8) and 0.25 volume of acetylacetone at 100 °C for 15 minutes. After that, the reagent mixture was cooled in an ice bath for 15 minutes. The modified Ehrlich’s reagent was added to the cooled mixture and stayed at room temperature for 30 minutes. The absorbance was determined at 554 nm using Epoch 2 microplate spectrophotometer (BioTek Instruments, USA).

### RNA Preparation and qRT-PCR

To prepare total RNA, the CGPB02 and CGPB04 strains were cultivated in modified CGXII medium and harvested at 12 h. Total RNA in the cell pellet was extracted using a Hybrid-R prep kit (GeneAll, Korea) according to the manufacturer’s instructions. Total RNA with random primer was denatured at 65 °C for 10 minutes, and reverse-transcriptionally synthesized to cDNA templates by M-MLV reverse transcriptase. All oligonucleotides for qRT-PCR were designed based on target gene sequence (Supplementary Table S2). qRT-PCR analysis was determined using a StepOnePlus thermocycler (Thermo Fisher Scientific, USA) with HiPi Real-Time PCR 2x Master Mix (SYBR green) according to the manufacturer’s instructions. The mRNA level of each gene of the CGPB04 strain was calculated relative to those of the CGPB02 strain using the comparative ΔΔCt method and was always normalized using the 16S rRNA level as an internal standard^62^.

## Electronic supplementary material

Supplementary information

## References

[1] Auwarter W, Ecija D, Klappenberger F, Barth JV (2015). Porphyrins at interfaces. Nat. Chem..

[2] den Boer D (2013). Detection of different oxidation states of individual manganese porphyrins during their reaction with oxygen at a solid/liquid interface. Nat. Chem..

[3] Mathew S (2014). Dye-sensitized solar cells with 13% efficiency achieved through the molecular engineering of porphyrin sensitizers. Nat. Chem..

[4] Yella A (2011). Porphyrin-sensitized solar cells with cobalt (II/III)-based redox electrolyte exceed 12 percent efficiency. Science.

[5] Ryu WH (2016). Heme biomolecule as redox mediator and oxygen shuttle for efficient charging of lithium-oxygen batteries. Nat. Commun..

[6] Carter KA (2014). Porphyrin-phospholipid liposomes permeabilized by near-infrared light. Nat. Commun..

[7] Zou Q (2017). Biological Photothermal Nanodots Based on Self-Assembly of Peptide-Porphyrin Conjugates for Antitumor Therapy. J. Am. Chem. Soc..

[8] Ussia M (2018). Freestanding photocatalytic materials based on 3D graphene and polyporphyrins. Sci. Rep..

[9] Yella A (2014). Molecular engineering of push-pull porphyrin dyes for highly efficient dye-sensitized solar cells: the role of benzene spacers. Angew. Chem., Int. Ed. Engl..

[10] Alibabaei L (2010). Application of Cu(II) and Zn(II) coproporphyrins as sensitizers for thin film dye sensitized solar cells. Energy Environ. Sci..

[11] Wu Y, Zhang Q, Liu JC, Li RZ, Jin NZ (2017). A novel self-assembly with two acetohydrazide zinc porphyrins coordination polymer for supramolecular solar cells. Org. Electron..

[12] Iritani K, Tahara K, Hirose K, De Feyter S, Tobe Y (2016). Construction of cyclic arrays of Zn-porphyrin units and their guest binding at the solid-liquid interface. Chem. Commun. (Cambridge, U. K.).

[13] Franke M (2016). Zinc Porphyrin Metal-Center Exchange at the Solid-Liquid Interface. Chemistry.

[14] Chen L, Bai H, Xu JF, Wang S, Zhang X (2017). Supramolecular Porphyrin Photosensitizers: Controllable Disguise and Photoinduced Activation of Antibacterial Behavior. ACS Appl. Mater. Interfaces.

[15] Hyeon JE (2018). Biomimetic magnetoelectric nanocrystals synthesized by polymerization of heme as advanced nanomaterials for biosensing application. Biosens. Bioelectron..

[16] Calvete, M. J. F. *et al*. A Cost-Efficient Method for Unsymmetrical Meso-Aryl Porphyrin Synthesis Using NaY Zeolite as an Inorganic Acid Catalyst. *Molecules* **22**, doi:10.3390/molecules22050741 (2017). 10.3390/molecules22050741 PMC6154588 28475140

[17] Henriques CA (2014). Ecofriendly Porphyrin Synthesis by using Water under Microwave Irradiation. ChemSusChem.

[18] Bosca F, Tagliapietra S, Garino C, Cravotto G, Barge A (2016). Extensive methodology screening of meso-tetrakys-(furan-2-yl)-porphyrin microwave-assisted synthesis. New J. Chem..

[19] Zhao EM (2018). Optogenetic regulation of engineered cellular metabolism for microbial chemical production. Nature.

[20] Shomar H (2018). Metabolic engineering of a carbapenem antibiotic synthesis pathway in *Escherichia coli*. Nat. Chem. Biol..

[21] Choi SY (2016). One-step fermentative production of poly(lactate-co-glycolate) from carbohydrates in *Escherichia coli*. Nat. Biotechnol..

[22] Hsu TM (2018). Employing a biochemical protecting group for a sustainable indigo dyeing strategy. Nat. Chem. Biol..

[23] Kadish, K. M., Smith, K. M. & Guilard, R. *The porphyrin handbook*. (Academic Press, 2000).

[24] Anttila J (2011). Is coproporphyrin III a copper-acquisition compound in *Paracoccus denitrificans*?. Biochim. Biophys. Acta, Bioenerg..

[25] Layer G, Reichelt J, Jahn D, Heinz DW (2010). Structure and function of enzymes in heme biosynthesis. Protein Sci..

[26] Georgi T, Rittmann D, Wendisch VF (2005). Lysine and glutamate production by *Corynebacterium glutamicum* on glucose, fructose and sucrose: roles of malic enzyme and fructose-1,6-bisphosphatase. Metab. Eng..

[27] Joo YC, Hyeon JE, Han SO (2017). Metabolic Design of *Corynebacterium glutamicum* for Production of l-Cysteine with Consideration of Sulfur-Supplemented Animal Feed. J. Agric. Food Chem..

[28] Joo, Y. C. *et al*. Bio-Based Production of Dimethyl Itaconate From Rice Wine Waste-Derived Itaconic Acid. *Biotechnol. J*. **12**, 10.1002/biot.201700114 (2017). 10.1002/biot.201700114 28846199

[29] Kim SJ, Hyeon JE, Jeon SD, Choi GW, Han SO (2014). Bi-functional cellulases complexes displayed on the cell surface of *Corynebacterium glutamicum* increase hydrolysis of lignocelluloses at elevated temperature. Enzyme Microb. Technol..

[30] Park, S. H. *et al*. Metabolic engineering of *Corynebacterium glutamicum* for L-arginine production. *Nat. Commun*. **5**, 10.1038/ncomms5618 (2014). 10.1038/ncomms5618 25091334

[31] Jo S (2017). Modular pathway engineering of *Corynebacterium glutamicum* to improve xylose utilization and succinate production. J. Biotechnol..

[32] Ramzi AB, Hyeon JE, Kim SW, Park C, Han SO (2015). 5-Aminolevulinic acid production in engineered *Corynebacterium glutamicum* via C5 biosynthesis pathway. Enzyme Microb. Technol..

[33] Ramzi AB, Hyeon JE, Han SO (2015). Improved catalytic activities of a dye-decolorizing peroxidase (DyP) by overexpression of ALA and heme biosynthesis genes in *Escherichia coli*. Process Biochem..

[34] Noh MH, Lim HG, Park S, Seo SW, Jung GY (2017). Precise flux redistribution to glyoxylate cycle for 5-aminolevulinic acid production in *Escherichia coli*. Metab. Eng..

[35] Feng L (2016). Metabolic engineering of *Corynebacterium glutamicum* for efficient production of 5-aminolevulinic acid. Biotechnol. Bioeng..

[36] Zhang J, Kang Z, Chen J, Du G (2015). Optimization of the heme biosynthesis pathway for the production of 5-aminolevulinic acid in *Escherichia coli*. Sci. Rep..

[37] Kwon SJ, de Boer AL, Petri R, Schmidt-Dannert C (2003). High-level production of porphyrins in metabolically engineered *Escherichia coli*: Systematic extension of a pathway assembled from overexpressed genes involved in heme biosynthesis. Appl. Environ. Microbiol..

[38] Nielsen MT (2015). Assembly of highly standardized gene fragments for high-level production of porphyrins in *E. coli*. ACS Synth. Biol..

[39] Choi SI, Park J, Kim P (2017). Heme Derived from *Corynebacterium glutamicu*m: A Potential Iron Additive for Swine and an Electron Carrier Additive for Lactic Acid Bacterial Culture. J. Microbiol. Biotechnol..

[40] Toriya M (1993). Zincphyrin, a novel coproporphyrin III with zinc from *Streptomyces* sp. J. Antibiot. (Tokyo).

[41] Wang L, Elliott M, Elliott T (1999). Conditional stability of the HemA protein (glutamyl-tRNA reductase) regulates heme biosynthesis in *Salmonella typhimurium*. J. Bacteriol..

[42] Wang L, Wilson S, Elliott T (1999). A mutant HemA protein with positive charge close to the N terminus is stabilized against heme-regulated proteolysis in *Salmonella typhimurium*. J. Bacteriol..

[43] Frunzke J, Gatgens C, Brocker M, Bott M (2011). Control of Heme Homeostasis in *Corynebacterium glutamicum* by the Two-Component System HrrSA. J. Bacteriol..

[44] Yu X, Jin H, Cheng X, Wang Q, Qi Q (2016). Transcriptomic analysis for elucidating the physiological effects of 5-aminolevulinic acid accumulation on *Corynebacterium glutamicum*. Microbiol. Res..

[45] Wennerhold J, Bott M (2006). The DtxR regulon of *Corynebacterium glutamicum*. J. Bacteriol..

[46] Brune, I. *et al*. The DtxR protein acting as dual transcriptional regulator directs a global regulatory network involved in iron metabolism of *Corynebacterium glutamicum*. *BMC Genomics* **7**, 10.1186/1471-2164-7-21 (2006). 10.1186/1471-2164-7-21 PMC1382209 16469103

[47] Bott M, Brocker M (2012). Two-component signal transduction in *Corynebacterium glutamicum* and other corynebacteria: on the way towards stimuli and targets. Appl. Microbiol. Biotechnol..

[48] Santamaria R, Gil JA, Mesas JM, Martin JF (1984). Characterization of an Endogenous Plasmid and Development of Cloning Vectors and a Transformation System in. Brevibacterium-Lactofermentum. J. Gen. Microbiol..

[49] Yamamoto S, Suda M, Niimi S, Inui M, Yukawa H (2013). Strain Optimization for Efficient Isobutanol Production Using *Corynebacterium glutamicum* Under Oxygen Deprivation. Biotechnol. Bioeng..

[50] Bentley WE, Mirjalili N, Andersen DC, Davis RH, Kompala DS (1990). Plasmid-encoded protein: the principal factor in the “metabolic burden” associated with recombinant bacteria. Biotechnol. Bioeng..

[51] Ramirez EA, Velazquez D, Lara AR (2016). Enhanced plasmid DNA production by enzyme-controlled glucose release and an engineered *Escherichia coli*. Biotechnol. Lett..

[52] Labbe RF, Vreman HJ, Stevenson DK (1999). Zinc protoporphyrin: A metabolite with a mission. Clin. Chem. (Washington, DC, U. S.).

[53] Gibson KD, Neuberger A, Tait GH (1962). Studies on the biosynthesis of porphyrin and bacteriochlorophyll by *Rhodopseudomonas spheroides*. 1. The effect of growth conditions. Biochem. J..

[54] Dailey HA, Gerdes S, Dailey TA, Burch JS, Phillips JD (2015). Noncanonical coproporphyrin-dependent bacterial heme biosynthesis pathway that does not use protoporphyrin. Proc. Natl. Acad. Sci. USA.

[55] Dailey TA (2010). Discovery and Characterization of HemQ An Essential Heme Biosynthetic Pathway Component. J. Biol. Chem..

[56] Dailey HA, Gerdes S (2015). HemQ: An iron-coproporphyrin oxidative decarboxylase for protoheme synthesis in Firmicutes and Actinobacteria. Arch. Biochem. Biophys..

[57] Celis AI (2017). A Structure-Based Mechanism for Oxidative Decarboxylation Reactions Mediated by Amino Acids and Heme Propionates in Coproheme Decarboxylase (HemQ). J. Am. Chem. Soc..

[58] Alenezi K, Tovmasyan A, Batinic-Haberle I, Benov LT (2017). Optimizing Zn porphyrin-based photosensitizers for efficient antibacterial photodynamic therapy. Photodiagn. Photodyn. Ther..

[59] Toriya M, Yamamoto M, Saeki K, Fujii Y, Matsumoto K (2001). Antitumor effect of photodynamic therapy with zincphyrin, zinc-coproporphyrin III, in mice. Biosci., Biotechnol., Biochem..

[60] Espinas NA, Kobayashi K, Takahashi S, Mochizuki N, Masuda T (2012). Evaluation of Unbound Free Heme in Plant Cells by Differential Acetone Extraction. Plant Cell Physiol..

[61] Mauzerall D, Granick S (1956). The occurrence and determination of delta-amino-levulinic acid and porphobilinogen in urine. J. Biol. Chem..

[62] Livak KJ, Schmittgen TD (2001). Analysis of relative gene expression data using real-time quantitative PCR and the 2(T)(-Delta Delta C). Methods (Amsterdam, Neth.).

