# Supplementary material for: Biosynthesis of organic photosensitizer Zn-porphyrin by diphtheria toxin repressor (DtxR)-mediated global upregulation of engineered heme biosynthesis pathway in Corynebacterium glutamicum
Source: Sci Rep. 2018 Sep 27;8:14460. doi: 10.1038/s41598-018-32854-9 (PMC6160403; doi:10.1038/s41598-018-32854-9)
Supplement: Supplementary file 1 — Supplementary information [file 41598_2018_32854_MOESM1_ESM.docx]

**[Supplementary information]**

# Biosynthesis of organic photosensitizer Zn-porphyrin by diphtheria toxin repressor (DtxR)-mediated global upregulation of engineered heme biosynthesis pathway in *Corynebacterium glutamicum*

Young Jin Ko^1^, Young-Chul Joo^1^, Jeong Eun Hyeon^1^, Eunhye Lee^1^, Myeong-Eun Lee^1^, Jiho Seok^1^, Seung Wook Kim^2^, Chulhwan Park^3^ & Sung Ok Han^1,*^

^1^ Department of Biotechnology, Korea University, Seoul 02841, Republic of Korea

^2^ Department of Chemical and Biological Engineering, Korea University, Seoul 02841, Republic of Korea

^3^ Department of Chemical Engineering, Kwangwoon University, Seoul 01897, Republic of Korea

^*^ Corresponding author

Department of Biotechnology, Korea University,

Seoul 02841, Republic of Korea

Tel: +82-2-3290-3151

Fax: +82-2-3290-3151

Correspondence and requests for materials should be addressed to S.O.H. (email: samhan@korea.ac.kr)

**
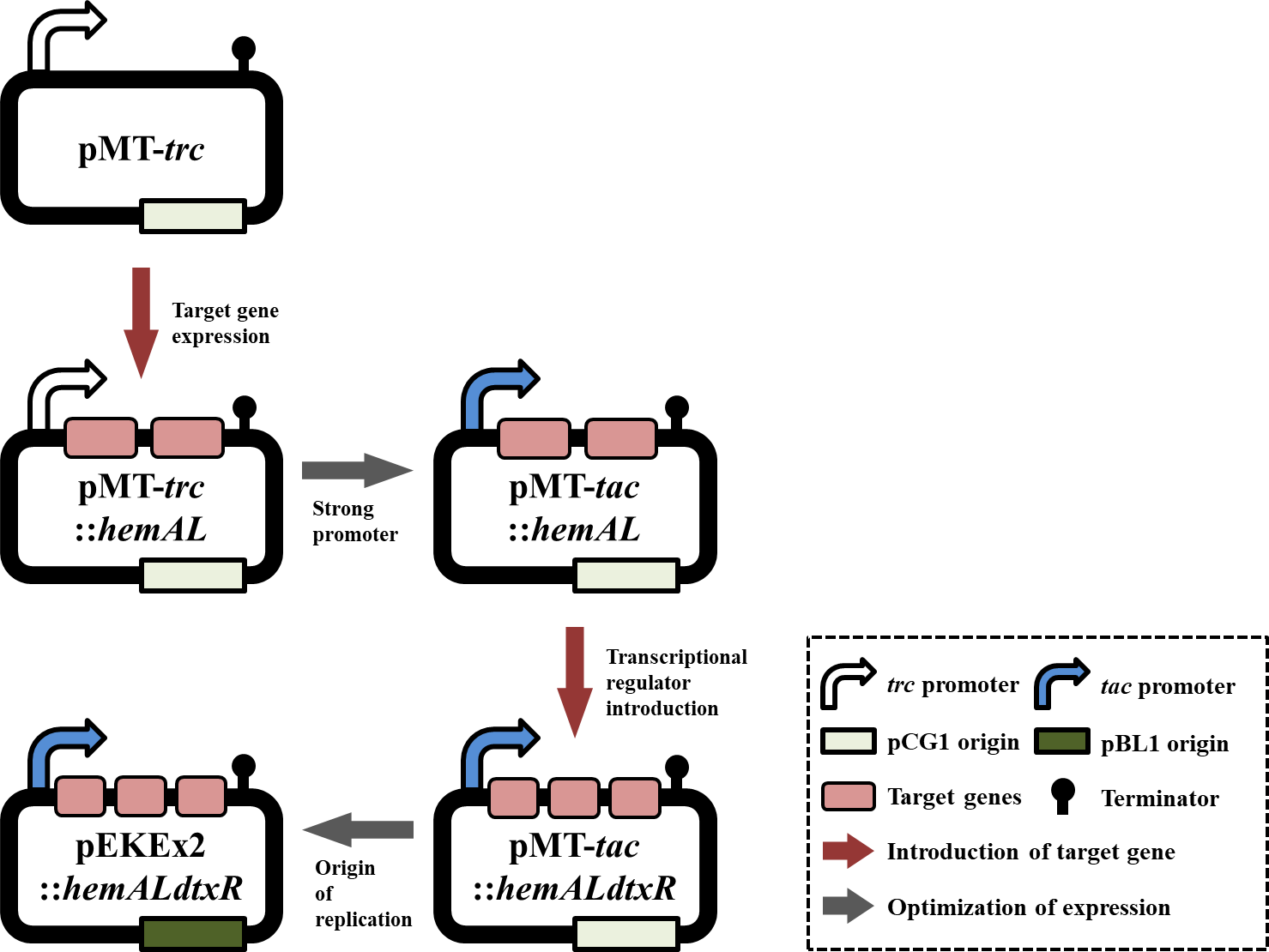
**

**Figure S1.** A schematic overview of genetic tools used in this study. Major symbols were described in dashed black line box.

**Figure S2.** ALA production and growth characteristics of the CGPB01 and CGPB02 strains after 72 h of shake-flask cultivation. The x-axis, left y-axis and right y-axis represent engineered strains, the titer of ALA and OD_600_, respectively. The cultivation of all engineered strains was conducted in triplicate, and all data and error bars denote mean values of three independent experiments and standard deviations of mean values, respectively.

**Figure S3.** Porphyrin production characteristics of the CGPB03, CGPB04, and CGPB07 strains during 72 h of shake-flask cultivation. The x-axis and y-axis represent engineered strains and the titer of porphyrins produced, respectively. The cultivation of all engineered strains was conducted in triplicate, and all data and error bars denote mean values of three independent experiments and standard deviations of mean values, respectively.

**Figure S4.** CP III and heme production in the CGPB04 strain in the culture condition of various iron sulfate concentrations. The x-axis, left y-axis and right y-axis represent the iron sulfate concentration and titer of CP III and heme produced, respectively. The cultivation of all engineered strains was conducted in triplicate, and all data and error bars denote mean values of three independent experiments and standard deviations of mean values, respectively. Symbols: orange color, CP III production; green color, heme production.

**Figure S5.** UV-Vis absorption spectra of metabolites produced in cultivation with or without zinc sulfate of the CGPB07 strain. The purple line represents optical characteristics of metabolites produced in zinc sulfate-supplemented cultivation. The orange line represents optical characteristics of metabolites produced in cultivation without zinc sulfate. The gray line represents optical characteristics of metabolites produced in cultivation without zinc sulfate. The x-axis and y-axis denote wavelength and absorbance unit measured in UV-Vis spectrophotometer, respectively.

**Figure S6.** Porphyrin production and growth characteristics of the ECPB01, ECPB02 and ECPB03 strains during 72 h of shake-flask cultivation. The x-axis, left y-axis and right y-axis represent engineered strains, the titer of porphyrins produced and OD_600_, respectively. The cultivation of all engineered strains was performed in the medium used in the research of Kwon *et al*. who have constructed the metabolically engineered *E. coli* for the porphyrin production^1^. This experiment was conducted in triplicate, and all data and error bars denote mean values of three independent experiments and standard deviations of mean values, respectively.

**Figure S7.** The analysis of mRNA expression levels of the *hemN* and *hemH* genes in the CGPB02 and CGPB04 strains by qRT-PCR. All engineered strains were incubated in CGXII medium with 4% glucose for 12 h. Relative mRNA expression levels of the *hemN* and *hemH* genes*,* measured in CGPB04 strain, were compared with those of the CGPB02 strain. The 16S rRNA level in all engineered strains was used as an internal reference for the normalization. The mRNA analysis of all engineered strains was conducted in triplicate, and all data and error bars represent mean values of three independent experiments and standard deviations of mean values, respectively.


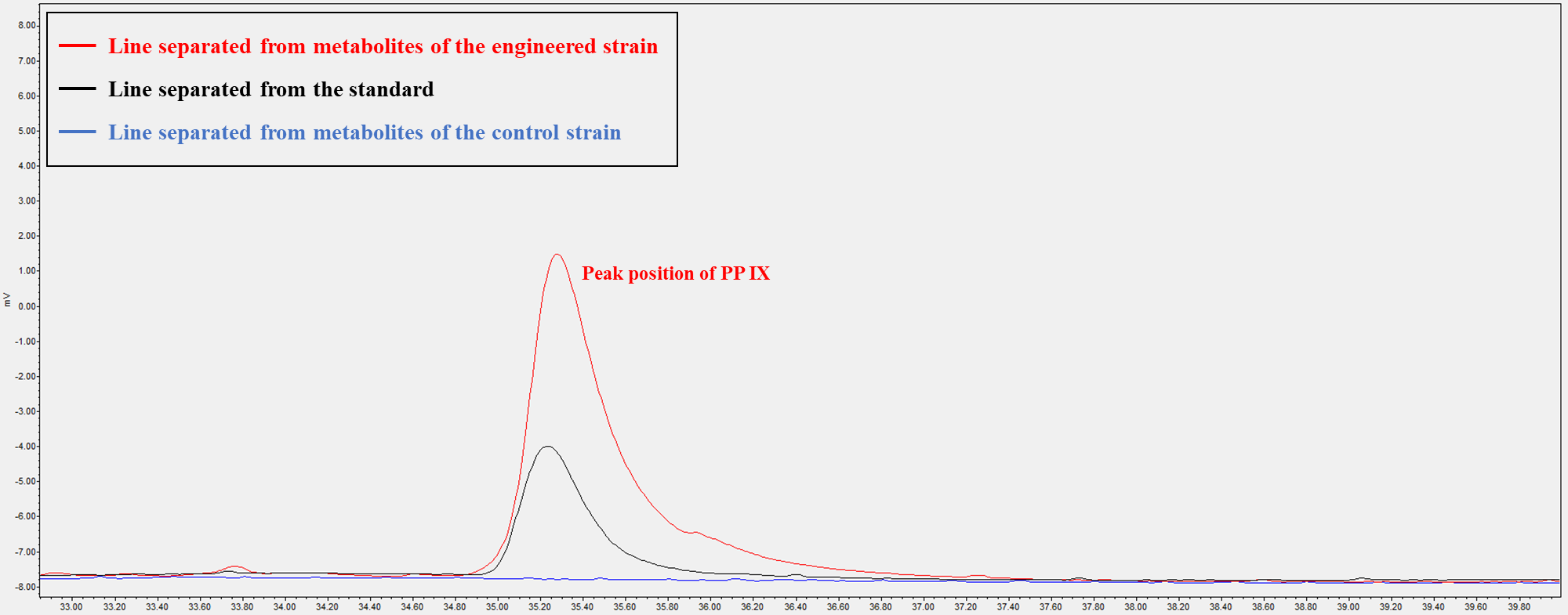


**Figure S8.** HPLC analysis of PP IX produced in the engineered *C .glutamicum* expressing the *hemF* (derived from *E. coli*) gene with the *hemA*, *hemL* and *dtxR* genes. PP IX produced in the engineered strain was shown on the same peak position (about 35.25 min) with the PP IX standard. In the peak data above, the red color line is the peak of PP IX produced in the engineered strain, and the black color line is the peak of PP IX standard, and the blue color line is the peak of supernatant produced in the control strain.

**Table S1.** List of oligonucleotides used in this study.

| **Primer name** | **Sequence (5'→3') *a*** |
| --- | --- |
| HemA BamHI F | gatGGATCCATGACCAAGAAGcttttagcgc |
| HemL NotI R | ATAGCGGCCGCtcacaacttcgcaaacacc |
| DtxR NotI F | AATGCGGCCGCAAGGAGATATACATGAAGGATCTGGTCGATACCACC |
| DtxR NotI R | AATGCGGCCGCTTAGCCCTCAACCTTTTCTACGCG |
| HemA PstI F | AATCTGCAGAAGGAGATATACATGACCAAGAAGCTTTTAGCGC |
| HemA SalI R | ACTGTCGACTCACAACTTCGCAAACACCC |
| DtxR SalI F | ACTGTCGACAAGGAGATATACATGAAGGATCTGGTCGATACCAC |
| DtxR BamHI R | ACTGGATCCTTAGCCCTCAACCTTTTCTACGC |

^a^ Restriction enzyme site and ribosomal binding site (RBS) denote red and green color letter, respectively.

**Table S2.** List of oligonucleotides used in this study for qRT-PCR.

| **Primer name** | **Sequence (5'→3')** |
| --- | --- |
| RT hemA F | GGAATACGATCAGCGAGCTT |
| RT hemA R | CTCGTGGCATGGACAAATCC |
| RT hemL F | GCGGTTACACTCAGCGTTCC |
| RT hemL R | TAGTGTCAGAAGTCTGAGCG |
| RT hemB F | CGATGACCTGCTGATTATGG |
| RT hemB R | GCCTGAGCAACAGCCATCTG |
| RT hemE F | GTTGCCTGAGTACAAGAAGG |
| RT hemE R | CAACGGCACCACAATGTCAG |
| RT hemN F | GTTGGACACCTCGTACCAAT |
| RT hemN R | TCCTTGATCGTGGAAGATGG |
| RT hemY F | AGGTGGAGTGATGGGTATTC |
| RT hemY R | GCGAAGACACCACAGTATCG |
| RT hemH F | CCTTGACCTGTGTGGAATAC |
| RT hemH R | TTGGTTCAGGATTCCTACGC |
| RT HrrA F | GATTTCACCGTGGCTTCTGA |
| RT HrrA R | TTGCGAACCGCCTGATGACT |
| 16s rRNA F | CCGGTACGGCTACCTTGTTA |
| 16s rRNA R | CCGTCACGTCATGAAAGTTG |

**Reference**

1 Kwon, S. J., de Boer, A. L., Petri, R. & Schmidt-Dannert, C. High-level production of porphyrins in metabolically engineered *Escherichia coli*: Systematic extension of a pathway assembled from overexpressed genes involved in heme biosynthesis. *Appl. Environ. Microbiol.* **69**, 4875-4883, doi:10.1128/Aem.69.8.4875-4883.2003 (2003).
